# Impacts of a Care Robotics Project on Finnish Home Care Workers’ Attitudes towards Robots

**DOI:** 10.3390/ijerph17197176

**Published:** 2020-09-30

**Authors:** Teemu Rantanen, Teppo Leppälahti, Jaakko Porokuokka, Sari Heikkinen

**Affiliations:** 1 Tikkurila Campus, Laurea University of Applied Sciences, Ratatie 22, 01300 Vantaa, Finland; sari.heikkinen@laurea.fi; 2 Hyvinkää Campus, Laurea University of Applied Sciences, Uudenmaankatu 22, 05800 Hyvinkää, Finland; teppo.leppalahti@laurea.fi; 3 The Finnish Innovation Fund Sitra, Itämerenkatu 11-13, 00181 Helsinki, Finland; Jaakko.porokuokka@sitra.fi

**Keywords:** attitude, care robot, elderly, home care

## Abstract

Technological advances in elderly care have been rapid, and the introduction of robots in care will be a topical issue in the near future. There has been little research into the possibility of influencing care workers’ attitudes towards robots by project activities, and how to make the change easier for work communities. This study focuses on a robotics project that took place in elderly and home care services in one municipality in Finland (total of 45 care workers). During the project, four robotics workshops and one extended pilot session were implemented. The study follows quasi-experimental settings, and it included two measurements (before and after project activities) and a control group, but no randomization. The data were collected by questionnaires and were analyzed statistically. The project under study brought about minor positive changes in home care workers’ attitudes towards the usefulness of care robots. In the final measurement, the difference between the test group and the control group was significant in the two dimensions of positive attitudes. The research supports the hypothesis that project activities can be used to influence home care workers’ attitudes towards robots. This can also facilitate the introduction of care robots in home care services. However, the construction of a technology-positive care culture is a long-term process, which requires training and development, technological development and strong strategic management at various levels.

## 1. Introduction

In many countries a rapid change in the population structure leads to an ageing population, which is mainly due to low fertility, low immigration rates and increasing longevity [1,2]. In 2020, about 23% of the whole population of Finland was aged 65 years or older. In the year 2070, the number is estimated to reach 33% [3]. The ageing population presents a challenge to health and social care systems, and there will be an enormous increase in health and social service use and expenditure in the near future that requires improvements in how care is organized. Functional limitations, chronic conditions and dementia become more frequent with increasing age, and make people more dependent on other people and services [1,4,5]. The ability to cope with the basic activities of daily living (ADL) and the instrumental activities of daily living (IADL) declines with increasing age, and is a common problem in people over the age of 85 [6].

In many countries, there has been an ongoing shift from institutional care to home care services. In Finland, during the past two decades, official guidelines from the central government have encouraged living at home as long as possible. Finland represents the typical Nordic welfare state tradition, and older people living in their own homes receive support, assistive devices, home services, home nursing, and support services, such as meals, cleaning, transport services, or other supportive social welfare services universally, regardless of their financial situation. In Finland, municipalities are responsible for organizing home care services. From 2016 to 2018, the number of customers using home care services increased by 4000 persons [7].

In order to meet the service needs that are likely to increase due to the demographic change, there is an ongoing shift from traditional care services to technologically oriented services, which benefits robotics and other technical devices, allowing care work to be more interactive and less occupied with basic care. In addition, care work suffers from a shortage of home care workers, and it needs to be transformed in order to become more attractive to young people as a career option. The evolution of robotics and high technology can be seen as a promising development to face these challenges [8,9]. The introduction of high technology and robots into the environment of home care is still novel. Home care work requires flexible and innovative service planning, including the ability to face new methods of organizing services using high technology and robotics. High technology has been designed with a variety of functions, such as telecommunications, stimulation programs, and providing social, medical, or physical assistance that benefits both the elderly and the home care workers [10,11]. The hindrance to utilize technology and especially robotics in care work in general is related to the ability to use them, and also to the nature of care work that is seen as interactive human-to-human work [12,13,14].

The introduction and adoption of a home healthcare robot can be studied from different points of view [15]. The theoretical starting points of the present study are based on discussions about attitudes towards robots [16,17,18,19,20,21,22,23,24,25]. Consistent with previous studies, this study assumes that workers’ attitudes play a key role in the success of robot deployment.

Numerous experiments using different robots and the evaluations of those experiments have been carried out in elderly care. The most studied robot is the Paro; a soft, seal-resembling therapy robot that incorporates a variety of sensors. In addition, telepresence robots which typically combine video calling and mobility capabilities, as well as social robots whose key feature is the ability to communicate through speech have been studied. The results for the Paro, in particular, have been positive for reducing depression in older adults [26]. According to the quasi-experimental study of Obayashi, Kodate and Masuyam [27], socially assistive robots also have great potential for improving older people’s quality of life. The telepresence robot has also proven to be easy to use, acceptable and useful [28,29]. A therapy robot (Paro), social robots, a telepresence robot, and some other robots (cf. Section 2.1) have been utilized in this study. However, the focus of the research is not so much on the benefits of robots for the elderly, but on changing the attitudes of the work community towards robots.

In previous studies, the duration of the intervention has typically been one or two single sessions, or a period of up to a few months [26,30]. Although short-term robotic experiments have been conducted and evaluated, there is a lack of research on the impacts of long-term robotics development in elderly care. Thus, instead of a single experiment, this study focuses on long-term development, and included several workshops and pilot experiments in elderly services carried out over four years.

The present study analyses the impacts of a care robotics project that took place in elderly care in a small municipality in southern Finland. According to previous studies, experiences with technology and robots increase the acceptance of robots [31,32]. Thus, the project under study sought to expose care workers to robots, and investigates what changes the project brought about in care workers’ attitudes towards robots and their usefulness. The changes are examined in relation to a control group. In this study, the term care robot is used in a broad sense, referring to any robot that is used for a care task, especially as a support for the independent living of the elderly or to assist in home care.

## 2. Background

### 2.1. Project Activities

The project Robots and the Future of Welfare Services (ROSE) (2015–2020) piloted various robots in the context of elderly care, and at the same time, developed the characteristics of robots to be better suited to this environment. In addition, the project examined the change brought about by the introduction of care robots from the perspective of service systems and work communities, as well as from an ethical point of view. The multidisciplinary consortium involved several universities and research institutes from Finland, and represented expertise in health and social care, humanities, and automation technology.

During the project activities, the home care workers and the elderly of the municipality were exposed to a variety of robots. Periods of short-term exposure and one extended pilot session were implemented in home care services by the project (see Table 1). Concurrent with the project, medicine dispensers placed in clients’ homes were also introduced in the home care facilities of the municipality.

In total, six workshops (see Table 1) took place during the project, but some were carried out two or three times so that as many employees as possible could participate in them. All members of the experimental group were invited to the workshops, but in practice, a maximum of 35 people participated.

**Figure 1:**
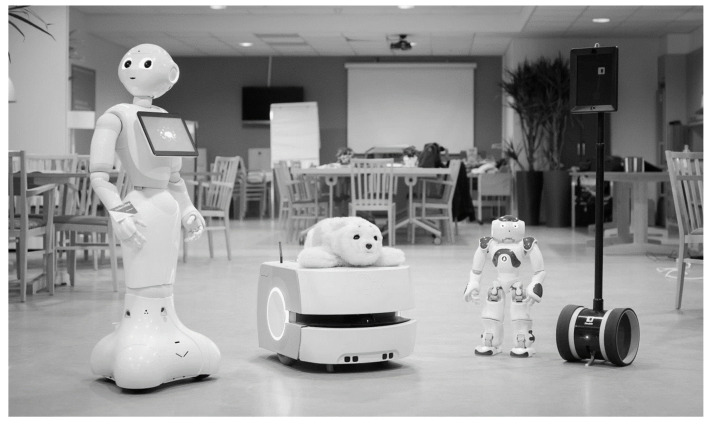
Robots used in the project (2nd robotics workshop in 2016).

The project began with a workshop aimed at outlining the work in home care services, its challenges for research design, and measure constructions. This was followed by four workshops conducted with workers and the elderly. The project used a telepresence robot (Double 2) and a social robot (Pepper). The second workshop used a total of five different robots (Figure 1).

An extended pilot placement of a robot was conducted in late 2018. The social robot Pepper was placed in housing services for a duration of five weeks. The robot featured three applications developed based on input from earlier research. The first application was designed to stimulate seniors’ memory through music and historical trivia from past decades. The second application allowed listening to the news from the Finnish national broadcasting company. The third application was an email-based messaging service utilizing face recognition. The services were entirely voice controlled. The robot was placed in a unit with 10 elderly people. Located in one of two living rooms, the clients were instructed to use the robot as they wished. The workers and clients were instructed on how to operate the robot, and technical support was provided when required. The use of the robot was documented using a locally installed camera. Following the pilot, the clients were interviewed individually, and the home care workers were interviewed as a focus group. At the end, the results were presented to the working group.

The elderly that took part to the workshops and robot pilot were all users of the care services and all had a service plan. They did not suffer from memory disorders, but they had other limitations that were mainly physical. Their condition was actively followed by the nurse and doctor. They all lived in the same rural-like area, far from the big cities, and as Finns their ethnic background was very homogeneous.

### 2.2. The Concept of Attitude

This study examines the impacts of the project from the perspective of a change in attitude. There is no generally accepted definition of the concept of attitude, but typically, attitude is defined as valuing a particular object [33]. The relationship between attitudes and behavior has been studied extensively, and for example, according to Ajzen [34], specific attitudes explain behavior much better than general attitudes. Thus, from the point of view of the introduction of care robots, care workers’ attitudes towards robots can be assumed to be more central than, for example, general attitudes towards technology.

Attitudes towards robots have also been studied from different perspectives [11]. First, attitudes have been studied at the societal level. For example, the European Commission’s Special Eurobarometer 382 [35] mapped public attitudes towards robots and their perceived usefulness in different areas of application, as well as their acceptance in different tasks in different countries in Europe. Second, attitudes have been studied from the perspective of emotional reactions related to robots and perceptions of robots [36,37,38]. In this presented study, the research focuses specifically on the attitudes that arise from immediate interactions with robots. Erebak and Turgut [25], in turn, have studied attitudes from the perspective of the trust evoked by seeing pictures of robots.

Nomura’s Negative Attitudes towards Robots Scale (NARS) [17] combines these two perspectives and focuses only on negative attitudes. The measure is divided into three dimensions. The first dimension relates to attitudes towards situations of interaction with robots (NARS1), the second to attitudes towards the social influence of robots (NARS2), and the third dimension to attitudes towards the emotions in interaction with robots (NARS3). Studies have revealed that there are cultural differences in attitudes towards robots [24]. For example, the Japanese prefer more human-like robots than Europeans [22]. However, the NARS scale (which was developed in Japan) can also be applied in Western countries [19].

In addition, attitudes towards the use of care robots in home care and in the support of independent living have been examined. Studies have shown that the elderly consider that robots are most useful specifically for detecting falls and providing emergency assistance [19,20]. Informal caregivers also see robots as useful reminders [20]. According to care workers, health care robots are useful, among other things for lifting heavy things, monitoring the location of people, turning lights and electrical appliances on/off, as well as being able to remind care workers and the elderly of things such as lunchtime or hydration levels [19]. According to a study by Alaiad and Zhou [21], home health care robots are considered useful for measuring the vital signs of the elderly on a regular basis, and recording or submitting them to a doctor. The robots are also useful assistants in monitoring patients and communications, as well as reminding patients to take their medications. The present study separately investigates all three dimensions of Nomura’s negative attitudes scale (NARS), as well as three different kinds of positive attitudes which address the promotion of safety, practical home care, and the provision of guidance and reminders [11].

Flandorfer’s [32] review shows the importance of sociodemographic factors in the acceptance of social robots. In particular, many studies reveal the significance of age, gender, education level, experience in technology and culture. On the other hand, Backonja et al. [23] have shown that older, middle-aged and younger adults have similar attitudes regarding the social impact of robots and their comfort with robots, as well as having similar negative attitudes towards robots.

All in all, there has been a lot of research on attitudes towards robots. In addition, individual robot experiments and their effects on customers have been studied. However, previous studies have mainly focused on short-term pilots using only one type of robot. This study focuses on a long-term work community development process that used a variety of robots.

## 3. Materials and Methods

### 3.1. Aim and Hypothesis

The present study focuses on a care robotics project carried out in municipal elderly services and especially in-home care services in a small municipality in southern Finland in 2015–2020. The study investigates the impact of project activities on care workers’ attitudes towards robots. Our main hypothesis is: (H) Project activities can be used to influence home care workers’ attitudes towards robots.

### 3.2. Settings

The present research follows a quasi-experimental setting, and focuses on a development project that was carried out in one municipality. As with experimental settings, the research process included a first measurement before the intervention, the intervention itself, and a second measurement. The intervention (the care robotics project) has been implemented in the test group, and the changes in the test group have been compared to changes in the control group. Randomization and blindness are, of course, not possible in such a research setting related to the development of the work community. The intervention was carried out in a municipality that was enthusiastic about cooperating with the research.

### 3.3. Sample

The data were collected by questionnaires in both the test group and the control group before and after the intervention. The test group covers employees of the home care work unit of the studied municipality, a total of 45 persons. The control group is formed in such a way as to represent the totality of Finnish care workers as well as possible. People working with clients, including physiotherapists, occupational therapists, and team leaders were included in both test and control groups, while managers and administrative secretaries were excluded.

### 3.4. Data Collection

The first data collection took place in March–May 2016, and the second data collection in November–December 2019. In the test group, the data was collected using a paper questionnaire. Participants filled in the form themselves during the development meetings (first workshop and meetings to present pilot results). In the control group, part of the data were collected for practical reasons by means of an electronic questionnaire. The practical arrangements for the questionnaires and requests for background information took place through home care supervisors by e-mail and telephone.

### 3.5. Measures

In the present study, negative attitudes are investigated using the Negative Attitudes towards Robots Scale (NARS), which consists of a total of 14 questions [17]. The measure divides into three sub-scales: attitudes towards human–robot interaction in specific situations, the social influences of robots, and the human–robot interaction and emotions related to it. A Finnish version of NARS was based on the English-language version [18]. The measure of positive attitudes is based on previous studies of attitudes towards care robots [19,20,21], and the development and validation of the measures took place as part of the research process. All questions were framed as statements with Likert-type scale response items (ranging from 1 = totally disagree to 5 = totally agree) (see Appendix A).

The face validation was carried out in focus group interviews of in-home care workers (8 groups), and by pre-testing among nursing students (*n* = 15). After data collection, answers were factorized (Principal Axis Factoring method, Varimax rotation with Kaiser Normalization), the internal consistency was investigated by Cronbach’s alpha, and relationships between sum variables were examined in light of the theory of planned behavior [34]. This validation process has been described in more detail in previous studies [11].

### 3.6. Analysis

The variables measuring attitudes were constructed by averaging the scores from the Likert-scale statements so that each variable consisted of three or four items. The internal consistency of the variables was investigated using Cronbach’s alpha coefficient, and the normality of the distributions was examined graphically and by looking at skewness and kurtosis coefficients. The actual analyses were performed parametrically.

The distribution of respondents in the test and control groups was compared by Student’s t-test and a chi-square test. The differences in attitudes between the localities included in the control group, were in turn analyzed by a one-way analysis of variance. Before the analyses, the assumption of homogeneity of variances was checked using Levene’s test. Age differences between the locations included in the control group were examined using the Brown–Forsythe test because the variances of the groups differed. Differences in educational level (practical nurses and other workers with an equivalent level of social and health education versus registered nurses and other workers with a higher education degree) between these locations were examined using the chi-square test. In order for the test conditions to be met, the smallest groups had to be combined during the test, however, this did not change the results.

Differences between the test group and the control group, as well as between the first and second samples, were analyzed using the Student’s t-test.

### 3.7. Ethical Issues

The study was conducted according to the instructions given by the Finnish National Board on Research Integrity [39] and the ethical principles of the WMA (World Medical Association) Declaration of Helsinki [40]. An ethical review for the performance and publication of the research was obtained from the FUAS (Federation of Universities of Applied Sciences) Advisory Board on Ethics (1.6.2016). Research approvals were granted by each organization that participated in the study. The respondents were informed about the voluntary nature, confidentiality and anonymity of their responses. In addition, the information letter contained the contact details of the researchers. Anonymity was guaranteed as the questionnaires were returned without any personal data.

## 4. Results

### 4.1. Respondents

The average age of the participants in the project was 43.5 years in 2016, and 46.5 years in 2019. The majority of them were women (94%). More than 60% of the respondents were practical nurses and about 20% were registered nurses (20.0% in first sample and 23.5 in second sample). (Table 2).

In term of the distribution of gender, there were only few differences between the respondent groups. In the second data set, the average age of the test group was higher than that of the control group, but the difference was not significant (t = 1.551, *p* = 0.123). In addition, there were differences in the respondents’ educational background, particularly in the second sample. The proportion of practical nurses was higher in the control group than in the test group, and the proportion of registered nurses was smaller. However, according to the chi-square test, there was no significant difference (χ^2^ = 2.31, *p* = 0.129) in the proportion of higher-educated persons (register nurses, social workers, etc.) between test and control groups. All in all, no significant differences in demographic variables can be found between the test group and the control group.

The response rate was quite good in the test group (87.5% and 75.6%) where data were collected during team development meetings. In contrast, response rates in the control group were significantly lower, at only 15.5% in the first and 11.8% in the second sample.

Respondents in the first and second questionnaires differed only slightly, but it is not possible to say reliably how many respondents took both surveys. 73.5% of the respondents in the test group had at least 3.5 years of work experience in home care services, and the majority of them also participated in the first survey.

### 4.2. Reliability of Measures

The reliability of the variables related to positive attitudes was quite high, except for one which was greater than 0.8. The alpha coefficients for the negative attitude variables were slightly lower. In the second sample, the reliability of the NARS3 variable was only α = 0.68 (see Table 3).

### 4.3. Analysis of the Control Group

The current state of robotics in home care services varied slightly between the localities selected for the control group. Security alarms were in use in all locations. Remote consultation has started at three locations, and medicine distribution robots are in use at two locations. In addition, different safety-related technologies were used in the localities and various robot pilot programs had been carried out.

The samples were collected from the same location in Finland. However, in the first and second sets of data, the regional distribution of the control group respondents differed. In addition, the data collection took place in slightly different ways (Table 4).

There are no significant differences in the ages of the respondents between the different localities included in the control group (First sample: df1 = 3, df2 = 110, F = 2.31, *p* = 0.080; second sample: df1 = 3, df2 = 105, F = 1.91, *p* = 0.132). In contrast, there were differences in the level of education of the respondents between localities (First sample: df = 2, χ^2^ = 17.2, *p* < 0.001; second sample: df = 2, χ^2^= 16.2, *p* < 0.001). In particular, the proportion of register nurses was higher in locality 4.

Next, the effect of control group locations in the second survey is analyzed. According to the analysis of variance, no significant difference was found in positive or negative attitudes (Table 5). Thus, it can be assumed that differences in the state of robotics and the distribution of respondents between samples do not substantially affect the results

### 4.4. Analysis of Differences in Attitudes

The results show that there were some differences between the test group and the control group even before the project started. Negative attitudes towards the emotions in interactions with robots were significantly stronger in the control group than in the test group. However, this difference disappeared during the project as the negative attitude strengthened in the test group and weakened in the control group. Overall, it seems that the project has not reduced the negative attitudes of care workers towards robots. Indeed, it would even appear to be the opposite, although the changes are not significant.

In contrast, positive attitudes towards the usability of care robots seem to have strengthened during the project. The changes are not statistically significant, but in the second survey, the test group and the control group differed significantly. The members of the test group were more positive about the view that the robots can provide practical assistance and can act as guides and prompters (Table 6).

There are differences in the proportion of nurses between different groups (see Section 4.3). However, the observed differences in positive attitudes between the test group and the control group is not explained by differences in the educational level of the respondents. In particular, the differences in positive attitudes are also significant among practical nurses and other workers with an equivalent level of social and health education (Robots as helpers in practical home care: t = 2.035, *p* = 0.044; Robots as guides and prompters: t = 1.994, *p* = 0.048).

## 5. Discussion

### 5.1. Findings

The present study focuses on a care robotics project that took place in municipal elderly services and especially in home care services in Finland. The study analyzed the impacts of the project based on care workers’ attitudes. The results of the study are ambiguous. At the end of the project, the perception of the usefulness of robots was significantly stronger in the test group than in the control group. Thus, the research supports the hypothesis that project activities can be used to influence home care workers’ attitudes towards robots. 

On the other hand, the strengthening of attitude in the test group was not statistically significant. Moreover, in this study, the effect was limited to positive attitudes. Negative attitudes related to robots could not be influenced by the project. Rather, it appears that during the project, negative attitudes strengthened slightly in the test group. However, specific positive attitudes related to perceived usefulness of care robots for different practical tasks can explain the adoption of care robots more strongly than the negative attitudes that do not relate to the context of home care or the supporting of older people [11]. Thus, the observed minor differences in positive attitudes can be considered important from the perspective of the introduction of care robots.

The project used a social robot (Pepper), and so is expected that the care workers in the test group found the robots useful in guiding and counselling the elderly. In contrast, it is surprising that workers in the test group placed more emphasis on the usefulness of robots in practical tasks than workers in the control group. For example, assistance in moving from bed to chair is quite a complex task to automate in real homes. Lifting robots like the bear robot Riba (Robot for Interactive Body Assistance) are, in practice, too large and heavy for private homes.

During the research process, the limited capacity of existing robots and the complexity of care work were observed [41]. Medicine dispensers and advanced security technologies have become more common in private homes, and various remote connectivity options are being tested and developed all the time. However, the large-scale use of robots in home care still requires a lot of technological development.

Attitudes towards robots have been studied quite a lot [16,17,18,19,20,21,22,23,24,25]. Similarly, a lot of short experiments have been done on the effectiveness of different robots in the care context [26,27,28,29]. The results of these studies have been largely encouraging. However, in contrast, less research has been done on how the attitudes of care employees towards the introduction of robots can be made more positive. Mast et al. [20] combined an attitude survey and a user-centered development of a human-robot interaction concept. Consistent with this, the present study emphasizes the perspective of user-driven development.

Vichitkraivin and Naenna [42] have shown that there are different resistance factors affecting the adoption of healthcare robot technology. The main barriers relate to the technology and its usability, the time required for deployment, and the perceived usefulness of change. This study focused on the change factor, but the idea was to reduce change resistance through a long-term process. However, this research makes visible the challenge of changing attitudes. It seems that especially the negative feelings and fears associated with robots are difficult to change. This result is surprising. During the workshops and during the pilot, the employees saw the robots and got to meet them, so one could have assumed that this would also reduce the fear and negative attitudes associated with them. However, this kind of change did not appear. At least in part, this is because the professionalism of care workers is largely built around providing personal support and encounters.

Bedaf et al. [43] have highlighted that the introduction of robots is not justified when tasks can be solved as effectively by simpler and cheaper technology. For example, robots may not be needed to increase the safety of the elderly, or to improve communication between the elderly and relatives. On the other hand, other perspectives on robots also emerged during the project. Even if robots do not always achieve concrete benefits, robots can also bring quality and variety to everyday life for both seniors and workers.

We can also ask what this study says on a more general level about the significance of project activities. In recent years, there have been a great number of community development projects focused on health technology in Europe. We can even talk about some kind of “project optimism”. However, according to the results of this study, there is little basis for this optimism. The construction of a technology-positive care culture is a long-term process. There are different care workers in the field of home care, and not all of them welcome technology with joy. It seems that it is not possible to create a new culture merely by using single projects, or even more comprehensive initiatives such as this project, where the home care workers had a possibility to ask questions, experience how the robots work, and discuss technological development in general. To be able to affect attitudes requires long-term activities and structures, which include training and development, technological development, and strong strategic management at various levels so that the users of the technology are active parts of the process. This may help to reduce suspicious attitudes towards robotics and other technological devices.

However, it is worth noting that the project benefitted the elderly care at home in ways beyond its research results. Robotization in home care services is still rare, and implementing robots in today’s home care is challenging. The project gave home care workers a chance to prepare for the time when robotization will be capable of reforming care services. The workers in home care services had a chance to get to know technological devices, and get an idea of how they work and how to work with them. It gave them an opportunity to consider important matters not only related to technological restrictions, but also issues related to the use of robotics in home care, such as legal and ethical dilemmas.

### 5.2. Limitations

The project under study has taken place in a particular time and locality, and cannot therefore be repeated in the same format in the future. As care robotics become more prevalent in elderly care, pilot experiments and exposure to care robots through long-term projects are not sufficient. However, in the future, automation technology is expected to provide more direct benefits either to clients or to work effectiveness.

The research design presents some challenges. First, the groups were not randomized. Second, the response rates remained low, especially in the control group, and the study could not confirm whether the respondents were the same persons in the first and second surveys. Third, the size of the test group was quite small, and partly due to this, no statistically significant change was observed in the study. Fourth, the study did not analyze the mechanisms that caused the differences between groups. These challenges are related to the fact that this is a pilot project carried out in real social and health organizations, and not an experimental study carried out under controlled conditions. On the other hand, this research is an example of how the effectiveness of development processes that take place in real elderly services can be evaluated. Unfortunately, however, the evaluation of development projects often remains at a level of measuring results and mapping opinions.

## 6. Conclusions

Previous studies have shown that experiences with technology and robots increase the acceptance of robots, and so, the psychological barriers associated with the introduction of robots can be overcome by exposing care workers to robots. However, the research reveals the challenging nature of home care work. It is very difficult to change care workers’ attitudes towards robots to be more positive and to make the implementation of care robots more acceptable in home care facilities, by way of project activities. This may be due to care workers’ fear that personal encounters with the elderly would be replaced by robots. Care workers need to experience the advantages robotics can bring to their own work and to the elderly. This challenges representatives of these types of projects to not only implement the care robots, but also to pay attention to the real advantages they can offer, and to share them as widely as possible. From the point of view of the introduction of robotics, education and content that are related to innovation and robotics play a key role.

## Figures and Tables

**Table 1:** Project activities.

Activity	Time	Participants	Actions in Workshops/Sessions	Robots Used ^1^	Data Collected
Face validation workshop	autumn 2015	35 home care workers	3 sessions and focus group interviews (total of 8 groups)	No robots	focus groups
1st robotics workshop	spring 2016	35 home care workers	3 sessions (total of 8 groups)	Double 2 ^1^	First survey before the robotics workshop
2nd robotics workshop	late 2016	5 elderly and 9 home care workers	one session	Double 2, Paro ^2^, Omron Lynx ^3^, Zora ^4^, Pepper ^5^	
3rd robotics workshop	early 2017	12 elderly and 2 home care workers	bingo session, Pepper as a host	Pepper	
4th robotics workshop	late 2017	9 elderly and 4 home care workers	exercise session, Pepper as a host	Pepper	
Pilot placement	late 2018, 5 weeks duration	unit with 10 elderly	memory stimulation through music and historical trivia, listening the news, an email-based messaging service utilizing face recognition	Pepper	
Workshop for final measurement	late 2019	22 home care workers	presentation of the results of the pilot (2 sessions)	Pepper	Second survey (absent workers completed the survey independently)

^1^ Double 2 is a two-wheeled, self-balancing videoconferencing robot for telepresence with lateral stability control, an audio kit, and a wide-angle camera kit. ^2^ Paro is a soft therapeutic robot in a shape of a pinniped animal with various sensors and the capability to learn in a way that the user prefers. It responds by moving its head and legs, and imitates the voice of a baby harp seal. ^3^ Omron Lynx is an autonomous indoor vehicle for transporting goods up to 60 kg in manufacturing, cleantech, warehousing, healthcare, etc., and can be used for custom applications, or utilized as a scalable fleet. ^4^ Zora (Zora robot Nao) is an interactive humanoid robot Nao equipped with the Zora application, which is a software making possible to operate robots without any specific coding or programming skills. Zora robot Nao recognizes speech, shapes, objects and people. It is bipedal small robot with two arms and touch sensors and it is used e.g., in eldercare and in healthcare. ^5^ Pepper is a social humanoid robot which is capable of recognizing faces and speech, and able to interact multimodally with the person talking to it or touching it.

**Table 2:** Samples.

Sample	Time	Group	N	Response Rate	Average Age (Years)	Share of Women	Share of Practical Nurses	Share of Register Nurses
1. Before intervention	March–May 2016	Test group	35	87.5%	43.5	94.3%	62.9%	20.0%
		Control group	165	15.5%	43.2	93.3%	57.6%	23.0%
2. After intervention	November–December 2019	Test group	34	75.6%	46.5	94.1%	61.8%	23.5%
		Control group	128	11.8%	42.7	94.5%	73.4%	10.9%

**Table 3:** Sum variables and their means, SD and reliability.

Variable	Items	Sample	*n*	Mean	SD	Cronbach’s *α*
NARS 1	4	before	200	2.79	0.93	0.799
		after	159	2.77	0.97	0.785
NARS 2	4	before	200	3.06	0.90	0.730
		after	162	3.20	0.91	0.738
NARS 3	4	before	200	3.92	0.91	0.795
		after	158	3.84	0.88	0.679
Robots as helpers in practical home care	4	before	199	2.58	1.05	0.824
		after	161	2.60	1.03	0.817
Robots as promoters of safety	4	before	199	3.38	1.09	0.865
		after	160	3.53	1.03	0.844
Robots as guides and prompters	3	before	199	3.78	0.97	0.817
		after	162	3.90	0.88	0.753

NARS: Negative Attitudes towards Robots Scale.

**Table 4:** The regional distribution of the control group respondents.

Location	First Sample (Before Intervention) *n* = 165		Second Sample (After Intervention) *n* = 128	
	Percentage of Respondents in the Control Group	How the Survey was Completed	Percentage of Respondents in the Control Group	How the Survey Was Completed
1 (medium-sized town)	24.2%	Electronically	39.8%	Paper form
2 (City)	21.8%	Electronically	20.3%	Electronically
3 (Small town)	5.5%	Electronically	7.8%	Electronically
4 (medium-sized town)	47.9%	Paper form	32.0%	Electronically
Missing information	0.6%		0.0%	
Total	100%		99.9%	

**Table 5:** Differences between localities in the control group. One-way analysis of variance.

Variable	df1	df2	*F*	*p*
NARS 1	3	121	1.24	0.297
NARS 2	3	124	1.64	0.183
NARS 3	3	121	0.49	0.688
Robots as helpers in practical home care	3	124	0.64	0.979
Robots as promoters of safety	3	123	0.08	0.970
Robots as guides and prompters	3	124	0.42	0.735

**Table 6:** Differences in attitudes between the test group and the control group in first and second samples.

Variable	Group	First Sample (Before Intervention)			Second Sample (After Intervention)			Difference		
		*n*	Mean	SD	*n*	Mean	SD	Change	t	*p*
NARS 1	Test group	35	2.557	0.784	34	2.522	0.833	0.035	−0.180	0.858
	Control group	165	2.842	0.958	125	2.834	0.992	−0.008	−0.073	0.942
	Difference, t(*p*)	−1.648 (*p* = 0.101)			−1.679 (*p* = 0.095)					
NARS 2	Test group	35	2.914	0.953	34	3.250	0.776	0.336	1.602	0.114
	Control group	165	3.091	0.884	128	3.191	0.939	0.100	0.939	0.349
	Difference, t(*p*)	−1.059 (*p* = 0.291)			0.334 (*p* = 0.738)					
NARS 3	Test group	35	3.543	0.835	33	3.765	0.927	0.222	1.040	0.302
	Control group	165	3.998	0.907	125	3.866	0.873	−0.132	−1.252	0.212
	Difference, t(*p*)	−2.735 (*p* = 0.007)			−0.583 (*p* = 0.561)					
Robots as helpers in practical home care	Test group	34	2.772	1.120	33	2.970	1.017	0.198	0.755	0.453
	Control group	165	2.535	1.038	128	2.506	1.017	−0.029	−0.238	0.812
	Difference, t(*p*)	1.197 (*p* = 0.233)			2.336 (*p* = 0.021)					
Robots as promoters of safety	Test group	34	3.353	0.962	33	3.750	0.855	0.397	1.785	0.079
	Control group	165	3.383	1.121	127	3.478	1.065	0.095	0.733	0.464
	Difference, t(*p*)	−0.147 (*p* = 0.883)			1.355 (*p* = 0.177)					
Robots as guides and prompters	Test group	35	3.971	0.729	34	4.245	0.648	0.274	1.646	0.104
	Control group	164	3.734	1.015	128	3.807	0.913	0.074	0.642	0.521
	Difference, t(*p*)	1.314 (*p* = 0.190)			2.623 (*p* = 0.010)					

## Appendix A. Statements Included in the Measures

**NARS 1**
I would feel uneasy if I was given a job where I had to use robots.
I would feel nervous operating a robot in front of other people.
I would hate the idea that robots or artificial intelligences were making judgements about things.
I would feel very nervous just standing in front of a robot.

**NARS 2**
Something bad might happen if robots developed into living beings.
I feel that if I depend on robots too much, something bad might happen.
I am concerned that robots would be a bad influence on children.
I feel that in the future society will be dominated by robots.

**NARS 3**
I would feel uneasy if robots really had emotions.
I would feel relaxed talking with robots.^1)^
If robots had emotions I would be able to make friends with them.^1)^
I feel comforted being with robots that have emotions.^1)^
^1)^ inverse item

**Robots as helpers in practical home care**
A care robot can help an older person with washing, dressing and using the toilet.
A care robot can help an older person move (e.g., moving from bed to chair).
A care robot can assist an older person in light household work (e.g., cooking, making their bed, doing the dishes, using the dishwasher).
A care robot can assist an older person in heavy household work (cleaning the windows, lifting).

**Robots as promoters of safety**
A care robot can help an older person communicate with relatives and friends.
A care robot can help observing an older person’s state of health (i.e., remote communication with doctor or nurse, real-time conveying of health information).
A care robot can help with medication (e.g., giving medicine, recognising medicine, observing medicine use).
A care robot can contribute to the safety of an older person living at home (e.g., by informing relatives and/or health care workers of a sudden change in the state of health).

**Robots as guides and prompters**
A care robot can remind an older person to take their medicine, of week days, of meetings,
A care robot can guide an older person with the use of phone or bank-related issues.
A care robot can guide an older person in physical exercise.
